# Factors associated with malaria infection among children after distribution of PBO-pyrethroid synergist-treated nets and indoor residual spraying in north-western Tanzania

**DOI:** 10.1371/journal.pone.0295800

**Published:** 2023-12-21

**Authors:** Ummi Abdul Kibondo, Jenny Renju, Eliud Lukole, Jacklin F. Mosha, Franklin W. Mosha, Alphaxard Manjurano, Mark Rowland, Natacha Protopopoff

**Affiliations:** 1 Vector Control Product Testing Unit (VCPTU) Ifakara Health Institute, Environmental Health, and Ecological Sciences, Bagamoyo, Tanzania; 2 Department of Epidemiology and Biostatistics, Institute of Public Health, Kilimanjaro Christian Medical University College (KCMUCo), Moshi, Tanzania; 3 The London School of Hygiene and Tropical Medicine, London, United Kingdom; 4 Kilimanjaro Christian Medical University College, Moshi, Tanzania; 5 National Institute for Medical Research, Mwanza Medical Research Centre, Mwanza, Tanzania; University of Glasgow College of Medical Veterinary and Life Sciences, UNITED KINGDOM

## Abstract

**Background:**

After a decade of successful control, malaria is on the rise again. The prevalence of malaria in Tanzania has increased from 7% in 2017 to 8% in 2022 and reached 18% in Kagera region in the North West of Tanzania. Malaria vectors in Muleba district Kagera have high level of pyrethroid resistance. The aim of this paper is to explore factors associated with malaria infection prevalence in children aged 6 months to 14 years in Muleba, where Long Lasting Insecticidal Net (LLIN) combining a pyrethroid insecticide and synergist piperonyl butoxide (PBO) that counteract resistance in the mosquitoes, was first distributed under trial conditions in 2015.

**Methods:**

The trial was a community randomized control in which there were two malaria prevalence cross-sectional household surveys each year (June and December) from 2015 to 2017 in Muleba. In this study we conducted a secondary data analysis of the December surveys only. Multilevel Poisson regression analysis was used to assess factors associated with malaria infection.

**Results:**

A total of 10,941 children and 4,611 households were included in this study. Overall malaria prevalence was 35.8%, 53.3% and 54.4% in the year 2015, 2016 and 2017 respectively. Living in an area with standard LLIN as opposed to the novel PBO synergist LLIN, being a male child, above 5 years of age, living in a house with open eaves, living in house without IRS, having head of household with no formal education, lower socioeconomic status and survey year were associated with increased risk of malaria infection.

**Conclusions:**

Using PBO LLIN reduced the risk of malaria infection. However, additional measures could further reduce malaria infection in areas of insecticide resistance such as housing improvement.

## Background

After a decade of successful control, malaria is on the rise again in Sub- Saharan Africa [1]. Between 2000 and 2015 Long Lasting Insecticidal Net (LLIN) were the most effective vector control strategy and accounted for 68% of malaria cases averted among the at-risk population [2] However, the emergence of insecticide resistance threatens the effectiveness of available LLINs [3].

In Tanzania, malaria accounts for 30% to 40% of all disease burden and 7% of all mortality [4]. Despite the scale-up of LLINs, the prevalence of malaria in Tanzania has increased from 7% in 2017 to 8% in 2022, a far cry from the national target of 5% by 2016 or the Tanzanian malaria control strategic plan of less than 1% prevalence by 2020 and sustainable development goal to end epidemic malaria by 2030 [5,6].

LLIN has been a core component of the Tanzanian malaria control strategy since 2009 [4,7]. Initiatives have included the Tanzania National Voucher Scheme (TNVS) and LLIN to pregnant women and infants, the NATNETS program [4,8], the National Malaria Control Program (NMCP) under-five catch-up campaign (U5CC) and in universal coverage campaign (UCC) [4]. In 2015 to 2016, over 22million LLINs were distributed through the UCC and schools net program (SNP) [9]. In addition to LLINs, indoor residual spraying (IRS) using lamdacyhalothrin 0.05%, then bendiocarb and finally pirimiphos methyl was conducted in the Lake Zone region with the support of President Malaria Initiative- PMI/USAID [9].

In 2008, Kagera region in North West Tanzania had the highest reported malaria prevalence (41%) across the country [4]. In 2012, after the scale-up of LLINs and IRS the prevalence decreased to 8% [4,10], however this trend reversed in 2016, with prevalence increasing again to 41% among children aged 6 to 59 months [5]. Malaria vectors in Tanzania, particularly Muleba district in Kagera region are highly resistant to pyrethroids [11–14]. Only 11% of *An*.*gambiae* died after exposure to pyrethroids [13]. In 2015, a cluster randomized controlled trial (RCT) was conducted with a novel LLIN to counteract the growing resistance [15]. PBO LLINs is a bi-treated net which incorporates permethrin insecticide and the synergist, piperonyl butoxide (PBO) while the standard (Olyset LLINs) LLIN is impregnated with permethrin only [16]. The trial was a success. One-year post distribution, malaria prevalence was lower in children with PBO LLIN (31%) than those with Standard LLIN (55%), (odds ratio 0.37; 95%CI: 0.21, 0.68). Two years post intervention prevalence was 46% in PBO LLIN arm and 68% in Standard LLIN arm (OR = 0.40; 95%CI: 0.20, 0.81) [15]. An effect was still observed for those that were using PBO LLIN on the third year [17]. Despite high coverage of Standard LLINs and PBO LLIN during the first two years the prevalence of malaria in Muleba was still high among children. Understanding the groups and individual at risk, factors associated with malaria infection in this area would be important to devise and deploy better vector control strategies. A secondary data analysis of the RCT data was conducted to explore factors associated with malaria infection among children aged 6 months to 14 years over the three years of the trial.

## Materials and methods

### Data source

The trial was a four arms, single-blinded, cluster randomized factorial design conducted in Muleba district in the northwestern part of Tanzania from 2015 to 2017. This trial is registered with ClinicalTrials.gov, number NCT02288637. The detail of the parent study procedures have been described elsewhere [15]. But in brief the study included 48 clusters which originated from 40 villages (larger villages were divided to form two clusters). The four study arms included different combination of vector control interventions implemented once in 2015:

1. Standard LLNs (Olyset ® Net, Sumitomo Chemicals, Japan)
2. PBO LLIN (Olyset ® Plus, Sumitomo Chemicals, Japan)
3. Standard LLIN with IRS (Actellic® 300CS, Syngenta, Switzerland)
4. PBO LLIN with IRS (Actellic® 300CS, Syngenta, Switzerland)

To assess the effectiveness of each intervention, household cross-sectional surveys were conducted in June and December of each year. A questionnaire was administered to obtain information on household and individual social demographic and economic factors including the availability and use of malaria prevention measures. Children aged 6 months to 14 years were tested for malaria infection using a rapid diagnostic test (CareStart Malaria HRP2/pLDH (pf/PAN) Combo, DiaSys, UK).

### Description of the sub- study

The cross-sectional data from December surveys was used for risks factors analysis over the three year’ life span of the LLINs. The type of LLIN was considered as the primary exposure therefore the arms receiving the same type of LLIN (Standard LLIN or PBO LLIN) were combined to form two arms regardless of IRS. Sample size was calculated for the first analysis of the RCT and is presented elsewhere [15]. A total of 10,941 children (5,475 from the PBO LLIN arm and 5,466 of children from standard LLIN arm) from 4,611 households were included in this study.

### Data management and statistical analysis

Data were extracted from the parent study database which was in Microsoft Access (Microsoft Corporation, Redmond, USA) format and transferred into STATA version13 (Stata-Corp, College Station, TX, USA) for further cleaning and statistical analysis. A description of all the independent variables included in the study is indicated in Table 1.

**Table 1:** Description of the study variables.

Independent Variable	Variable description
Age (Years)	Age of the child, categorised into under 5, 5- <10 and ≥10 years
Sex	Sex of the child
LLIN user	Binary variable ‘Yes’ if children reported to slept under LLIN the night before the survey. For this study, this was reported by the parent or guardian who was interviewed
Household socioeconomic status (SES)	Calculated as a weighted sum of data on household possessions and utilities, using principal components analysis and the scores divided into 5 quartiles. Elements included in the measure: electricity, radio, mobile phone, motorbike, bicycle, own farmland, livestock and household crowdedness (relative number of people per room). A total of 28 (0.6%) households missing some elements of SES were replaced with mean scores
Head of household education level	Categorised into 3 groups: No schooling, Primary and Secondary and Above
Improved housing structure	Binary variable, coded ‘Yes’ if the house has any of the following: iron roof, intact ceiling, full cement or tiles floor, bricks wall, plastered walls and ‘No’ if otherwise
Eaves of the house	Binary variable ‘open’ if the household has open eaves or ‘closed’ if the household has no open eaves
Indoor Residual Sprayed (IRS) in 2015	Binary variable ‘Yes’ if the household was sprayed in 2015
An animal kept inside the house	Binary variable ‘Yes’ if domestic animals are sleeping inside the household
LLIN ownership	Binary variable ‘Yes’ if the household owns at least one LLIN
LLIN Access	Binary variable ‘Yes’ if households have enough LLIN per sleeping places, was calculated by taking the number of available LLIN in the household divided by the number of sleeping places
Study arm	Based on the two types of LLIN distributed by the parent study, which considered as primary exposure:(Standard LLIN and PBO LLIN arm)
Survey Year	Includes the three survey rounds: 2015,2016 and 2017 which represent 9 months, 21 months and 33months post-LLIN distribution respectively

For categorical variables, numbers and proportions (percentages) in each level were calculated. For continuous variable median and inter-quartile range (IQR) were calculated.

Given that malaria prevalence was more than 40% in the study area; Poisson regression was used as an alternative to logistic regression to determine factors associated with malaria infection. Poisson regression estimates risk ratio (prevalence ratio) instead of odd ratio. For rare outcome (less than 10%) odds ratio approximates prevalence ratio or risks ratio, that’s why odds are used to present the risk. However when the outcome is very common odds tend to overestimate the risk [18–20].

A Multilevel modelling technique was used to estimate the risk ratio, given the hierarchical nature of the data (as the children are nested within a household and households are nested within clusters/villages). Household and village level variation in malaria prevalence was assessed from a null multilevel model that included only a random intercept term. The likelihood showed no variability at the household level; hence only village/cluster level was considered as random effect during this analysis. Multilevel mixed-effect generalized liner mixed model (meglm) with a Poisson distribution (family) and log link function was used in Stata.

A step-wise by backward elimination method was used for all factors associated with malaria infection with a p-value of 0.2 in the univariate model into a multivariable multilevel Poisson regression model. Interactions between study arm with LLIN usage and survey rounds were investigated. Interaction between household IRS status and survey round was also included. Akaike Information Criteria (AIC) was used to assess the model with and without interaction. The between cluster variability and Intracluster correlation coefficient (ICC) were also calculated and presented. Complete case analysis was considered.

## Ethics statement

The main trial was approved by the ethics review committees of the Kilimanjaro Christian Medical University College, the London School of Hygiene & Tropical Medicine, and the Tanzanian Medical Research Coordinating Committee (NIMR/HQ/R.8a/VolIX/1803). Written informed consent from parents or guardians was obtained for each survey. Ethical clearance to perform secondary analysis was obtained from the Kilimanjaro Christian Medical University College Research and Ethics Committee, Tanzanian (No.2119). Participant’s confidentiality was maintained by ensuring the use of participant identification number during data extraction and analysis.

## Results

### Participants and household characteristics

**Fig 1:**
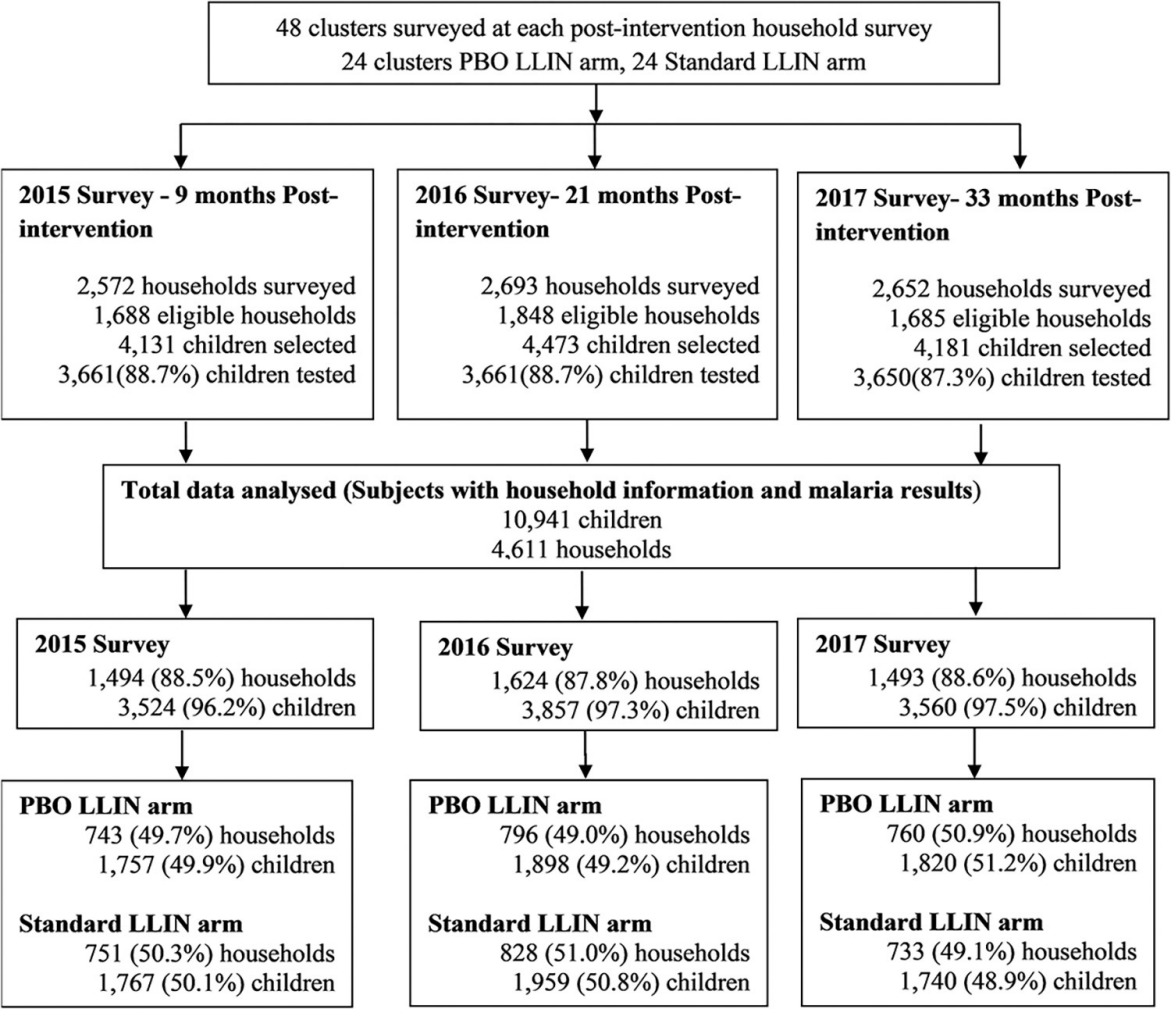
Flow chart of selected households and children included in the study, Muleba Tanzania.

A total of 48 clusters were surveyed at each time point. For the three surveys, data were available for 5,221 households with children aged 6 months to 14 years and 11,275 children were tested for malaria. About 11% of selected children in each survey year were not tested for malaria infection. After data cleaning and merging, 88% of all eligible households and 97% of selected children in each survey were analysed (Fig 1). The average number of children per cluster for the three surveys was 228.

A total of 10,941 children with a median age of 7 (IQR: 4, 10) years were analysed. There were an almost equal number of children between study arms across all surveys. LLIN usage dropped over survey time. However, the proportion was almost balanced between arms, except during the second year of the trial in 2016, 1221 (62.3%) of children in standard LLIN arm use LLIN and 1086 (57.2%) in PBO LLIN arm. A total of 4,611 households were analysed and were equally distributed between arms and almost half of the households in each arm were sprayed in 2015. Overall, within each survey year, LLIN ownership and access were almost similar between the two arms, but there was a drop over time (Table 2).

**Table 2:** Household and individual characteristics of study participants by study arm for 2015–2017 surveys, Muleba Tanzania.

Variable	2015 Survey		2016 Survey		2017 Survey	
	PBO LLIN	Standard LLIN	PBO LLIN	Standard LLIN	PBO LLIN	Standard LLIN
*Individual characteristics*, *n (%)*						
**Age group (age in years)**						
<5	570 (32.4)	570 (32.3)	575 (30.3)	567 (28.9)	523 (28.7)	472 (27.1)
5 to <10	674 (38.4)	665 (37.6)	748 (39.4)	775 (39.6)	686 (37.7)	642 (36.7)
10 to 14	513 (29.2)	532 (30.1)	575 (30.3)	617 (31.5)	611 (33.6)	626 (36.0)
**Sex**						
Male	878 (50.0)	900 (50.9)	941 (49.6)	955 (48.7)	879 (48.3)	840 (48.3)
Female	879 (50.0)	867 (49.1)	957 (50.4)	1004 (51.3)	941 (51.7)	900 (51. 7)
**LLIN user**	1404 (79.9)	1440 (81.5)	1086 (57.2)	1221 (62.3)	939 (50.2)	849 (48.8)
*Household characteristics*, *n (%)*						
**Household SES**						
Poorest	162 (21.8)	170 (22.6)	181 (22.7)	205 (24.8)	170 (22.4)	130 (17.7)
Poorer	157 (21.1)	165 (22.0)	147 (18.5)	157 (19.0)	154 (20.3)	140 (19.1)
Poor	143 (19.2)	148 (19.7)	166 (20.9)	153 (18.5)	151 (19.9)	173 (23.6)
Less Poor	171 (23.0)	177 (23.6)	174 (21.9)	189 (22.8)	139 (18.3)	140 (19.1)
Least Poor	110 (14.8)	91 (12.1)	128 (16.1)	124 (15.0)	146 (19.2)	150 (20.5)
**Head of household educational level**						
No schooling	195 (26.2)	199 (26.5)	213 (26.8)	234 (28.3)	186 (24.5)	168 (22.9)
Primary	505 (68.0)	528 (70.3)	553 (69.5)	566 (68.4)	542 (71.3)	530 (72.3)
Secondary and above	40 (5.4)	21 (2.8)	28 (3.5)	24 (2.9)	28 (3.7)	33 (4.5)
Missing	3 (0.4)	3 (0.4)	2 (0.3)	4 (0.5)	4 (0.5)	2 (0.3)
**Improved housing structure**	641 (86.3)	624 (83.1)	709 (89.1)	724 (87.4)	677 (89.1)	677 (92.4)
Missing	0	1 (0.1)	2 (0.3)	0	1 (0.1)	1 (0.1)
**House with an open eave**	455 (61.2)	500 (66.6)	455 (57.2)	481 (58.1)	376 (49.5)	379 (51.7)
**Animals kept inside the house**	126 (17.0)	122 (16.2)	176 (22.1)	180 (21.8)	148 (19.5)	165 (22.5)
Missing	3 (0.4)	1 (0.1)	0	0	0	0
**A household with ≥1 LLIN**	729 (98.1)	733 (97.6)	727 (91.3)	773 (93.4)	568 (74.7)	532 (72.6)
**A household with enough LLIN**	655 (88.2)	680 (90.5) *	550 (69.1)	600 (72.5)	431 (56.7)	424 (57.8)
**Household sprayed in 2015**	374 (50.3)	371 (49.4)	402 (50.5)	412 (49.8)	389 (51.2)	378 (51.6)
**A household with ≥1 study LLIN**	721 (97.0)	728 (96.9)	677 (85.1)	729 (88.0)	400 (52.6)	408 (55.7)
**A household with enough study LLIN**	628 (84.5)	656 (87.4) *	387 (48.6)	476 (57.5)	164 (21.6)	172 (23.5)

*Two households (0.3%) missing.

### Malaria prevalence and factors associated with malaria infection

The malaria prevalence increased over the years from 35.8% in 2015 to 54.4% in 2017. Overall malaria prevalence was 41.6% in the PBO LLIN arm and 51.6% among children living in the standard LLIN arm (Table 3).

**Table 3:** Univariate multilevel Poisson regression of factors associated with malaria infection among children in Muleba, Tanzania.

Variable	Total	Malaria Infection n (%)	Crude RR [95%CI]	P-value
**Age group (in years)**				
<5	3277	1312 (40.0)	1	
5 to <10	4190	2163 (51.6)	1.28 [1.20, 1.37]	<0.001
10 to 14	3474	1783 (51.3)	1.28 [1.19, 1.38]	<0.001
**Sex**				
Male	5393	2703 (50.1)	1	
Female	5548	2555 (46.1)	0.92 [0.87, 0.97]	0.003
**LLIN user**				
Yes	6939	3207 (46.2)	1	
No	4002	2051 (51.2)	1.15 [1.08, 1.21]	<0.001
**Household SES**				
Poorest	2253	1218 (54.1)	1	
Poorer	2175	1091 (50.2)	0.93 [0.85, 1.98]	0.074
Poor	2246	1080 (48.1)	0.89 [0.83, 0.98]	0.011
Less Poor	2406	1092 (45.4)	0.84 [0.78, 0.92]	<0.001
Least Poor	1861	777 (41.8)	0.80 [0.73, 0.87]	<0.001
**Head of household education level**				
Non-formal	2712	1409 (52.0)	1	
Primary	7786	3701 (47.5)	0.93 [0.88, 0.99]	0.026
Secondary and above	400	130 (32.5)	0.68 [0.57, 0.82]	<0.001
**Improved house structure**				
Yes	9696	4571 (47.1)	1	
No	1233	683 (55.4)	1.12 [1.03, 1.21]	0.007
**Eaves of the house**				
Closed	4746	2065 (43.5)	1	
Open	6189	3191 (51.6)	1.13 [1.06, 1.19]	<0.001
**Animal kept inside the house**				
No	8725	4104 (47.0)	1	
Yes	2206	1150 (52.1)	1.06 [0.99, 1.14]	0.051
**Study Arm**				
Standard LLIN	5466	2978 (54.5)	1	
PBO LLIN	5475	2280 (41.6)	0.74 [0.58, 0.95]	0.019
**A household with ≥1 LLIN**				
Yes	9704	4611 (47.5)	1	
No	1237	647 (52.3)	1.11 [1.02, 1.20]	0.014
**Household with enough LLIN**				
Yes	7975	3746 (47.0)	1	
No	2962	1509 (50.9)	1.09 [1.03, 1.16]	0.002
**Household IRS in 2015**				
Yes	5534	2407 (43.5)	1	
No	5407	2851 (52.7)	1.16 [0.90, 1.49]	0.249
**Survey year**				
2015	3524	1263 (35.8)	1	
2016	3857	2057 (53.3)	1.47 [1.37, 1.58]	<0.001
2017	3560	1938 (54.4)	1.52 [1.41, 1.64]	<0.001

In the univariate multilevel Poisson regression analysis, all factors except for the household IRS status had a statistically significant association with malaria infection among the selected children (Table 3).

In the multivariable multilevel analysis, about 5% (ICC = 0.05) of the total variability in the risk of malaria infection was attributed to the cluster/village. Based on the empty model (A model with outcome and cluster effect, before adding other variables) 6% of the variability in risk of malaria was due to clustering.

Findings from the multivariable multilevel Poisson analysis suggest age of the child, sex, household IRS status in year 1, household SES, head of house education, study arm and survey year remain significantly associated with risk of malaria infection after adjusting for other factors (Table 4).

**Table 4:** Multivariable multilevel Poisson regression analysis of factors associated with malaria infection among children in Muleba, Tanzania(N = 10,880).

Variable	Adjusted RR [95% CI] *	P-value
**Age group (in years)**		
<5	1	
5 to <10	1.27 [1.18, 1.36]	<0.001
10 to 14	1.26 [1.18, 1.36]	<0.001
**Sex**		
Male	1	
Female	0.91 [0.87, 0.97]	0.001
**Standard LLIN arm**		
LLIN user	1	
Non LLIN user	1.02 [0.94, 1.10]	0.652
**PBO LLIN arm**		
LLIN user	1	
Non LLIN user	1.08 [0.99, 1.19]	0.057
**Household SES**		
Poorest	1	
Poorer	0.96 [0.88, 1.04]	0.298
Poor	0.94 [0.86, 1.02]	0.149
Less Poor	0.90 [0.83, 0.98]	0.018
Least Poor	0.85 [0.77, 0.94]	0.002
**Head of household education level**		
Non-formal	1	
Primary	0.97 [0.91, 1.03]	0.316
Secondary and above	0.77 [0.64, 0.92]	0.005
**2015 survey**		
Standard LLIN arm	1	
PBO LLIN arm	0.68 [0.53, 0.88]	0.003
Households with IRS	1	
Households without IRS	1.47 [1.14, 1.89]	0.003
**2016 Survey**		
Standard LLIN arm	1	
PBO LLIN arm	0.70 [0.54, 0.90]	0.003
**2017 Survey**		
Standard LLIN arm	1	
PBO LLIN arm	0.80 [0.62, 1.03]	0.085
**Eaves of the house**		
Closed	1	
Open	1.12 [1.05, 1.19]	<0.001
**Random Effects**		
Between Cluster Variance	0.16	
ICC	0.05	

*Risk ratio adjusted for all variables in the table.

The age of the child was highly associated with malaria infection, children aged 5 to 10 years and above 10 years had 27% and 26% higher risk respectively compared to those less than 5 years of age with (aRR = 1.27; 95%CI: 1.18, 1.36) and (aRR = 1.26; 95%CI: 1.18, 1.36). Girls had a 9% lower risk of having malaria infection than boys (Table 4).

Children in the least poor household SES had a 15% lower risk compared to those in the poorest SES. On the other hand, households whose head had secondary or higher education level, children were less likely to have malaria infection as compared to those with no education. Living in the house with open eaves was associated with a 12% increased risk of infection compared to those in the house with closed eaves (Table 4).

Children in the PBO LLIN arm had 32% lower risk of malaria infection compared to those living in the standard LLIN arm in the year 2015, 30% lower risk in 2016 and 20% lower risk of having malaria infection in 2017. However, the difference was not statistically significant (aRR = 0.80; 95%CI: 0.62, 1.03). In 2015 survey, children in a household without IRS had a 47% higher risk of malaria infection (Table 4).

## Discussion

The results show an overall increase in malaria prevalence in the study area over the years. Prevalence was higher amongst children living in areas with standard LLINs as opposed to those living in villages with PBO LLINs. Other factors associated with malaria infection were being a male and greater than 5 years old. Children living in households of lower socio-economic status, those with open eaves, house without IRS and whose head of household had no formal education had increased risk of malaria infection.

The observed increase in malaria prevalence over the years could be explained by a decrease in mosquito net usage and in PBO concentration in the PBO LLIN. LLINs usage dropped from 80% to 50%. Findings from the same randomized control trial by Protopopoff et al. [15], reported that the PBO content in the LLINs had reduced from 9.5 g/kg at 0 month to 1.6g/kg after 21 months of use. Also, from the same trial it was reported that, after three years of use permerthrin content for both nets were reduced by 55% while 97% of the PBO content was lost [21]. Furthermore, the physical integrity of the nets over the 3 years may have been compromised due to use and washing practices. In Tanzania, 39% of LLINs were reported to be “too torn” according to WHO criteria 2 to 4 years of use [7]. Whilst in Rwanda 58% of the LLINs fell into “need for replacement” category, two years post distribution [22]. Studies have reported that, protective efficacy of the nets were reduced with deterioration of the nets and increasing hole numbers [23] especially in area where malaria vectors are resistant to insecticide [24]. Additionally, it has been shown that both net types were not found to last for three years in this area with median survivor of 1.9 years for the standard LLIN and 1.6 years for PBO net [21]. Thus, individuals were remained unprotective for a substantial period of time hence increase the chance of contracting malaria.

Throughout the study period, children living in the area with standard LLINs as opposed to those living in villages with PBO LLINs were more likely to have malaria infection regardless of LLIN usage. Data from the same trial demonstrated that after 20 months of use, there were less mosquitoes blood fed inside PBO nets compared to the standard LLINs. Suggesting that despite development of holes PBO LLINs provided better personal protection than standard LLINs arm [25]. This could indicates that standard LLIN might have lost some effectiveness due to mosquito resistance [15,26]. Similar results have been reported in the same study area in 2013; LLINs users had similar risk to get malaria as non-users [27]. A multi-country study in Benin, Cameroon, India, Kenya, and Sudan, showed that standard LLINs still offered protection regardless of insecticide resistance [28]. However, these countries had lower insecticides resistance levels as compared to the present study area.

Inconsistent with other reports [29,30], the present study showed that LLIN usage at individual level was not a significant factor in either study arms, after adjusting for other factors. Similar findings have been reported elsewhere [31,32]. This could be explained by the community protection LLIN offer beyond that of the individual user. In the present study net usage was high (80.7% and 60.1%) in the first and second year respectively. At high coverage, in addition of providing a protective barrier to the sleeper, LLIN reduced the density and lifespan of malaria parasite and therefore malaria infection [33–35]. Based on modelling done by Killen et al. (2007), a net usage as small as 35% could provide protection even to those who did not sleep under the LLINs. The data predicted that 75% of net usage can lead to 98% and 90% protection between net users and non-users respectively [36]. Another explanations is that “net usage” is gathered by inquiring from individuals/caregiver if they slept under the net the previous night [37]. However, this practice has some limitations as self-reported responses might be affected by a social desirability bias [37]. As a result, the study might over or underestimated the association between the real net usage practices in the community and risk for malaria infection. In this study this bias was minimized by observing if the mosquito net was correctly hung in the room during the household surveys. Effectiveness of the net could also be compromised by their physical condition [7,23]. This could be the case in the last survey year of this study were after the nets had been used for 30 months.

Similar to other studies [2,15,38,39], this study found a significant association between indoor residual spray and risk for malaria infection within one-year post-IRS. The effect of the pirimiphos-methyl spray on malaria reduction was observed up to 12 months’ post-intervention. The effect of IRS is not observed after the second year which is not surprising as IRS is usually done annually [4].

In line with other studies, this study also found that lower household SES [24,26,40–42], house with open eaves [43–45], low education level of household head [30,40], being male child and older children (above 5 years) [24,41,46,47] were associated with increased risk of malaria infection. A house with open eaves allows easy entrance and escape of mosquitoes, which expose residents at high risk of malaria infection [48]. Outdoor activities of the elder children or boys [47] and mosquito feeding behavior could influence this finding, as mosquitoes have been reported to bite during early evening hours (before bedding time) in Tanzania [49] and Zambia [50] after the introduction of vector control measures. In Eastern part of Tanzania, households with highest economic status were approximately 4 times more likely to replaced “too-torn” nets by new one [51]. This indicate that, households with lower SES are more likely to use old and torn net, hence increasing the risk of infection. Additional vector control measures, such as house improvements (closing eaves, screening doors and windows) [48,52] could be considered to further reduce the burden of malaria.

Other studies have reported important environmental factors which contribute to malaria infection such as land cover, land surface temperature and precipitation [42,52,53]. A limitation of this study is that we have not collected those factors.

## Conclusions

The present analysis suggests that using of PBO LLINs reduced the risk of malaria infection in this area of high pyrethroid resistance compared to Standard LLIN. However, the overall prevalence still remains high and increased during the 3 years of the trial. This suggest increasing coverage of the PBO LLINs as well as educational and communication campaigns to promote appropriate and consistent use of LLINs.

Furthermore, to lower prevalence and meet the national target, additional measures such as house improvements (closing eaves, screening doors and windows) and programs are needed to protect children in the household with poor SES, house with open eaves, low head education level and older children.

## Supporting information

S1 Checklist STROBE statement—checklist of items that should be included in reports of observational studies. (DOC)
