# Supplementary material for: Factors associated with malaria infection among children after distribution of PBO-pyrethroid synergist-treated nets and indoor residual spraying in north-western Tanzania
Source: PLoS One. 2023 Dec 21;18(12):e0295800. doi: 10.1371/journal.pone.0295800 (PMC10734997; doi:10.1371/journal.pone.0295800)
Supplement: S1 Checklist — (DOC) [file pone.0295800.s001.doc]

STROBE Statement—checklist of items that should be included in reports of observational studies

|  | Item No | Recommendation |
| --- | --- | --- |
| **Title and abstract** | 1 | (*a*) Indicate the study’s design with a commonly used term in the title or the abstract  **Please refer to page 2, lines 36-39.** |
|  |  | (*b*) Provide in the abstract an informative and balanced summary of what was done and what was found  **Provided in page 2-3, lines 26-48.** |
| Introduction | | |
| Background/rationale | 2 | Explain the scientific background and rationale for the investigation being reported  **Please refer to page 3-4, lines 50-85.** |
| Objectives | 3 | State specific objectives, including any prespecified hypotheses  **Explained in detail in the background page 4, lines 80-85.** |
| Methods | | |
| Study design | 4 | Present key elements of study design early in the paper  **Description of the parent study and current study have been explained under the subheading data source and description of sub study. Please refer to page 5-6, lines 89-110.** |
| Setting | 5 | Describe the setting, locations, and relevant dates, including periods of recruitment, exposure, follow-up, and data collection  **Description of the parent study and current study have been explained under the subheading data source and description of sub study. Please refer to page 5-6, lines 89-110.** |
| Participants | 6 | *Cross-sectional study*—Give the eligibility criteria, and the sources and methods of selection of participants |
|  |  | **Description of the parent study and current study have been explained under the subheading data source and description of sub study. Please refer to page 5-6, lines 89-110.** |
| Variables | 7 | Clearly define all outcomes, exposures, predictors, potential confounders, and effect modifiers. Give diagnostic criteria, if applicable  **Explained in page 5, page 6 (lines 107-110) and page 7, lines 135-140.**  **Also, in Table 1: Description of the study variables (Page 19)** |
| Data sources/ measurement | 8* | For each variable of interest, give sources of data and details of methods of assessment (measurement). Describe comparability of assessment methods if there is more than one group  **Elaborated in page 5-6 and Table 1 in page 19** |
| Bias | 9 | Describe any efforts to address potential sources of bias **NA** |
| Study size | 10 | Explain how the study size was arrived at  **Sample size and power sub section in page 6, lines 111-118.** |
| Quantitative variables | 11 | Explain how quantitative variables were handled in the analyses. If applicable, describe which groupings were chosen and why  **Included in page 6, lines 124- 125 and Table 1, page 19.** |
| Statistical methods | 12 | (*a*) Describe all statistical methods, including those used to control for confounding  **Refer to sub heading Data management and statistical analysis in page 6-7, lines 120-140.** |
|  |  | (*b*) Describe any methods used to examine subgroups and interactions  **Explained in page 7, lines 135-139.** |
|  |  | (*c*) Explain how missing data were addressed  **Page 7, line 140 and Table 1 in page 19.** |
|  |  | (*d*) *Cohort study*—If applicable, explain how loss to follow-up was addressed. **NA**  *Case-control study*—If applicable, explain how matching of cases and controls was addressed. **NA**  *Cross-sectional study*—If applicable, describe analytical methods taking account of sampling strategy  **Explained in page 7, lines 129-134.** |
|  |  | (*e*) Describe any sensitivity analyses **NA** |

| Results | | |
| --- | --- | --- |
| Participants | 13* | (a) Report numbers of individuals at each stage of study—eg numbers potentially eligible, examined for eligibility, confirmed eligible, included in the study, completing follow-up, and analysed  **Results section in page 8, figure 1 and Table 2.** |
|  |  | (b) Give reasons for non-participation at each stage**. Results section in page 8 and figure 1** |
|  |  | (c) Consider use of a flow diagram. **Figure 1** |
| Descriptive data | 14* | (a) Give characteristics of study participants (eg demographic, clinical, social) and information on exposures and potential confounders  **Results section: Participants and household characteristics page 8 and Table 2 in page 20** |
|  |  | (b) Indicate number of participants with missing data for each variable of interest  **Example: Head of household educational level (Table 2, Page 20)** |
|  |  | (c) *Cohort study*—Summarise follow-up time (eg, average and total amount) **NA** |
| Outcome data | 15* | *Cohort study*—Report numbers of outcome events or summary measures over time **NA** |
|  |  | *Case-control study—*Report numbers in each exposure category, or summary measures of exposure **NA** |
|  |  | *Cross-sectional study—*Report numbers of outcome events or summary measures  **indicated in page 9 and table 3 in page 21** |
| Main results | 16 | (*a*) Give unadjusted estimates and, if applicable, confounder-adjusted estimates and their precision (eg, 95% confidence interval). Make clear which confounders were adjusted for and why they were included  **Explained in page 9, Table 3 and Table 4 (Page 21-22)** |
|  |  | (*b*) Report category boundaries when continuous variables were categorized  **Refer to page 9, Table 3 and Table 4** |
|  |  | (*c*) If relevant, consider translating estimates of relative risk into absolute risk for a meaningful time period **NA** |
| Other analyses | 17 | Report other analyses done—eg analyses of subgroups and interactions, and sensitivity analyses  **Refer to page 10 and Table 4 in page 22** |
| Discussion | | |
| Key results | 18 | Summarise key results with reference to study objectives  **First paragraph in the discussion section refer to page 10, lines 197-202** |
| Limitations | 19 | Discuss limitations of the study, taking into account sources of potential bias or imprecision. Discuss both direction and magnitude of any potential bias  **Please refer to page 11-12, lines 230-236** |
| Interpretation | 20 | Give a cautious overall interpretation of results considering objectives, limitations, multiplicity of analyses, results from similar studies, and other relevant evidence  **Page 10-13** |
| Generalisability | 21 | Discuss the generalisability (external validity) of the study results  **Page 13, lines 258-266** |
| Other information | | |
| Funding | 22 | **NA** |

*Give information separately for cases and controls in case-control studies and, if applicable, for exposed and unexposed groups in cohort and cross-sectional studies.

**Note:** An Explanation and Elaboration article discusses each checklist item and gives methodological background and published examples of transparent reporting. The STROBE checklist is best used in conjunction with this article (freely available on the Web sites of PLoS Medicine at http://www.plosmedicine.org/, Annals of Internal Medicine at http://www.annals.org/, and Epidemiology at http://www.epidem.com/). Information on the STROBE Initiative is available at www.strobe-statement.org.
